# Seasonality Is the Main Determinant of Microbial Diversity Associated to Snow/Ice around Concordia Station on the Antarctic Polar Plateau

**DOI:** 10.3390/biology12091193

**Published:** 2023-08-31

**Authors:** Gerardo A. Stoppiello, Claudia Coleine, Ralf Moeller, Caterina Ripa, Daniela Billi, Laura Selbmann

**Affiliations:** 1 Department of Ecological and Biological Sciences, University of Tuscia, 01100 Viterbo, Italy; stoppiello@unitus.it (G.A.S.); cripa@unitus.it (C.R.); selbmann@unitus.it (L.S.); 2 Aerospace Microbiology Research Group, Radiation Biology Department, Institute of Aerospace Medicine, German Aerospace Center (DLR e.V.), D-51103 Cologne, Germany; ralf.moeller@dlr.de; 3 Department of Natural Sciences, University of Applied Sciences Bonn-Rhein-Sieg, D-53359 Rheinbach, Germany; 4 Department of Biology, University of Rome Tor Vergata, 00133 Rome, Italy; 5 Mycological Section, Italian Antarctic National Museum (MNA), 16128 Genova, Italy

**Keywords:** Antarctic Polar Plateau, extremophiles, life detection, fungi, bacteria, amplicon sequencing, extraterrestrial analogue

## Abstract

**Simple Summary:**

The Antarctic Polar Plateau is one of the most extreme environments on Earth and our knowledge on the microbial diversity inhabiting this region is still limited. The BacFinder project investigated microbial diversity on the snow surface of the Polar Plateau, focusing on the vicinity of the Concordia Antarctic Research Station, to assess the microbial diversity and the potential impact of human presence on such a pristine environment. We found that seasonality was the main driver for both bacterial and fungal assemblages, while biodiversity appeared unaffected by distance from the base. Amplicon sequencing revealed a predominance of *Basidiomycota* (49%) and *Ascomycota* (42%) for the fungal component. *Bacteroidota* (65.8%) is the main representative of the bacterial component. *Basidiomycetes* are almost exclusively represented by yeast-like fungi. Overall, the study highlighted the impact of human activity on the microbial composition in this environment and may provide critical information on the habitability of extra-terrestrial analogs on our planet and on the possibility to explore the surfaces of icy worlds.

**Abstract:**

The French–Italian Concordia Research Station, situated on the Antarctic Polar Plateau at an elevation of 3233 m above sea level, offers a unique opportunity to study the presence and variation of microbes introduced by abiotic or biotic vectors and, consequently, appraise the amplitude of human impact in such a pristine environment. This research built upon a previous work, which explored microbial diversity in the surface snow surrounding the Concordia Research Station. While that study successfully characterized the bacterial assemblage, detecting fungal diversity was hampered by the low DNA content. To address this knowledge gap, in the present study, we optimized the sampling by increasing ice/snow collected to leverage the final DNA yield. The V4 variable region of the 16S rDNA and Internal Transcribed Spacer (ITS1) rDNA was used to evaluate bacterial and fungal diversity. From the sequencing, we obtained 3,352,661 and 4,433,595 reads clustered in 930 and 3182 amplicon sequence variants (ASVs) for fungi and bacteria, respectively. Amplicon sequencing revealed a predominance of *Basidiomycota* (49%) and *Ascomycota* (42%) in the fungal component; *Bacteroidota* (65.8%) is the main representative among the bacterial phyla. *Basidiomycetes* are almost exclusively represented by yeast-like fungi. Our findings provide the first comprehensive overview of both fungal and bacterial diversity in the Antarctic Polar Plateau’s surface snow/ice near Concordia Station and to identify seasonality as the main driver of microbial diversity; we also detected the most sensitive microorganisms to these factors, which could serve as indicators of human impact in this pristine environment and aid in planetary protection for future exploration missions.

## 1. Introduction

Aerial transport by the atmospheric circulation has been identified as an important source of biological inputs to remote locations such as the Antarctic [1]. Yet, at continental sites, katabatic winds, flowing off the high continental plateau towards the coast, may inhibit local aerobiological transfer of propagules towards inland sites. The French–Italian Concordia Research Station, located on the Antarctic Polar Plateau, 3233 m above sea level (a.s.l.), is an extreme and the most isolated habitat of earth, offering a unique opportunity to study the presence, variation, and ability to perpetuate microbes introduced, by chance, through abiotic or human vectors, and to evaluate the impacts in such a pristine environment. In fact, microorganisms inhabiting the Antarctic ice sheet may have evolved mechanisms to withstand sub-zero temperatures, low temperatures, high solar UV radiation, osmotic pressures, and limited nutrient availability [2,3]. Moreover, the harshest conditions characterizing the Polar Plateau makes this location a suitable analogue for some extraterrestrial conditions [4]; thus, untangling microbial diversity, inhabiting one of the most extreme environments on Earth, may inform us on the terrestrial habitability but also on the possibility of life elsewhere in the Solar System, particularly in the subsurface of icy worlds [5]. The present research is based on a recent study performed by Napoli and colleagues [6], which, in the frame of the BacFinder project (European Space Agency), aimed to explore the microbial diversity of the surface snow surrounding the Concordia Research Station and potential human contamination. That study corroborated the use of DNA-sequencing-based techniques for revealing microbial presence in this remote environment and, for the first time, amplicon sequencing was leveraged to investigate both prokaryotic and eukaryotic microbial diversity of surface snow samples. In particular, despite the extracted metagenomic DNA being below the detection limit for all samples, it was possible to amplify and sequence the bacterial 16S rRNA gene for all samples; on the other hand, the eukaryotic 18S rRNA was amplified for a few only, while ITS was totally unsuccessful, hampering the detection of fungal diversity. To overcome the problem encountered by Napoli and co. [6] regarding the paucity of microbial DNA, significantly affecting the following high-throughput sequencing analyses, we here first optimized the sampling by increasing the ice/snow collected by up to 50%, leveraging the possibility to sequence specific microbial (i.e., fungi) groups. Previous attempts to determine what factors may influence microbial communities have largely focused on the relative importance of temperature and nutrient concentrations [7]. Our data gave, for the first time, a complete overview of both fungal and bacterial diversity associated with the surface snow/ice of the Antarctic Polar Plateau surrounding the Concordia Station emphasizing the effect of distances and seasonality on the variation of transient or resident microbes. Moreover, we individuated the most susceptible microbial compartment to these factors to be potentially used as indicators of the impact of human activities in such a pristine environment.

## 2. Materials and Methods

### 2.1. Study Area

**Figure 1:**
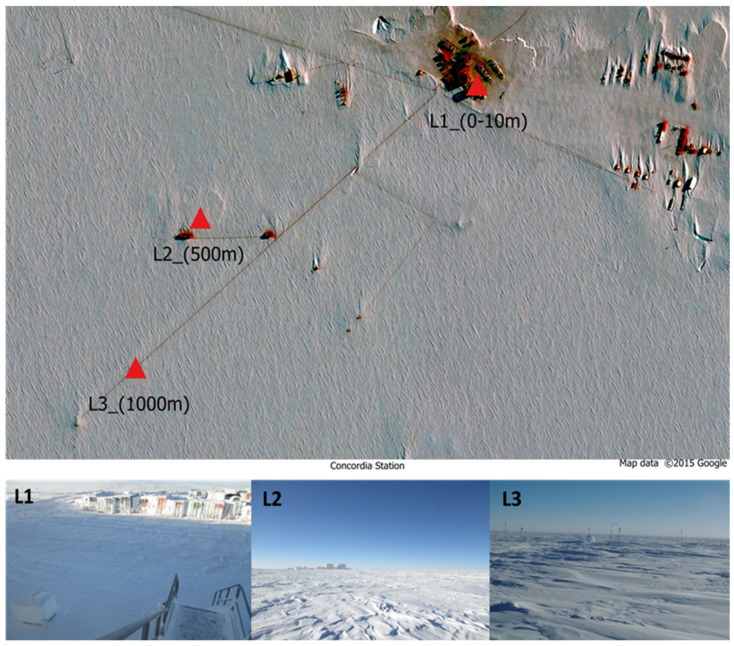
Map of the three sampling sites at different distances from Concordia Station. Area (L1) just outside; area (L2) 500 m; area (L3) 1000 m.

The ice samples were collected in the sampling area at the Concordia Research Station (75°06′01.8″ S 123°21′03.8″ E) (Figure 1), a French–Italian research facility built in a place called Dome C on the Antarctic Plateau, at 3233 m altitude. 

Temperatures can drop as low as −80 °C in winter, with an annual average of −50 °C. The closest human beings were about 600 km away at the Russian base of Vostok. Concordia is more remote than the International Space Station. The Concordia base accommodates up to 80 people during the austral summer, including technicians, logisticians and researchers. During the winter, a small group of 13 people, called the ‘Winter Over’, is confined for at least 9 months.

The harvesting of the ice samples investigated in this study was carried out from December 2018 to December 2019 at the Concordia Station. Fifteen 50 mL falcons containing snow were collected every month and at each distance. Summer ice samples were collected during the November–March period; winter ice samples were collected during the April–October period. Sampling was performed at 3 different distances from the Concordia Base, in proximity to the Concordia base (0–10 m, L1), medium distance from the base (500 m. L2), and in an area relatively far from the base (1000 m, L3) (Figure 1). The samples were then shipped to the University of Tuscia (Viterbo), where they were stored at −20 °C until downstream analyses. Sampling procedures are listed in further detail in Table S1.

### 2.2. DNA Extraction and Amplicon Sequencing

A total of 39 samples were processed for amplicon sequencing (3 distances for 13 months) (Table S1). The ice samples were gradually melted at 4 °C. The 15 aliquots of each sample were combined into a single pool (Table S1) and filtered using a 250 mL filtration apparatus (VWR^®^ Vacuum Filtration 0.2 µm) with a 0.2 µm porosity membrane. The filters were cut using a sterile scalpel and placed in 15 mL falcons and were then stored at −80 °C while pending DNA extraction.

Prior to DNA extraction, the membranes were crushed under sterile conditions in liquid nitrogen. Genomic DNA was extracted using the following extraction protocol using the Cetyltrimethyl ammonium bromide (CTAB) method [8,9], following the manufacturer’s instructions, and finally eluted in 45 µL. The extracted DNA was quantified using the Qubit dsDNA High Sensitivity Assay Kit (Life Technologies, Carlsbad, CA, USA) according to the protocol provided by the manufacturer. The V4 variable region of the 16S rDNA and Internal Transcribed Spacer (ITS1) rDNA was used to assess bacterial and fungal diversity, respectively. This variable region was amplified using primers F515 (GTGCCAGCMGCCGCGGTAA)/R806 (GGACTACHVGGGTWTCTAAT) for 16S as described by Caporaso et al. (2012) [10]. To amplify the variable region of ITS, primers ITS1F (CTTGGTCATTTAGAGGAAGTAA)/ITS2 (GCTGCGTTCTTCATCGATGC) were used as in Smith and Peay (2014) [11]. The PCR reactions were performed with a total volume of 25 μL, containing 1 μL of each primer (5 picomoles/μL), 12.5 μL of Taq DNA Polymerase (Thermo Fisher Scientific, Waltham, MA, USA), 9.5 μL of nuclease-free water (Sigma-Aldrich, Gillingham, UK), and 5 ng of template DNA. For the bacteria (16S rDNA), the amplification of the V4 variable region was performed following the following protocol: initial denaturation at 94 °C for 3 min, 35 cycles of denaturation at 94 °C for 45 s, annealing at 50 °C for 1 min, extension at 72 °C for 90 s, followed by a final extension at 72 °C for 10 min. For the fungal component (ITS1), amplification of the variable region was performed as follows: initial denaturation at 93 °C for 3 min, 35 cycles of denaturation at 95 °C for 45 s, annealing at 50 °C for 1 min, extension at 72 °C for 90 s, followed by a final extension at 72 °C for 10 min (Bio-Rad, Hercules, CA, USA).

The amplicon pool was sequenced in pair ends (2 × 300 bp) using the Illumina MiSeq platform. DNA amplification, quantification, purification, and sequencing were performed by the Edmund Mach Foundation (San Michele all’Adige, Italy).

### 2.3. Bioinformatics

From sequencing, raw reads were obtained: short synthetic DNA sequences. These reads, already demultiplexed by the Edmund Manch Foundation, were analyzed using AMPtk: Amplicon ToolKit for Next Generation Sequence data software v1.2.1 [12], a bioinformatics software that processes NGS amplicon data using USEARCH ([13] and VSEARCH [14]. The reads were trimmed, resulting in sequences with a length of 250 bp, discarding reads less than 100 bp in length, and chimera removal was performed by utilizing USEARCH with default parameters v. 9.2.64. Sequence quality filtering was performed with the expected error parameter of <1.0 [12]. The dataset was clustered with DADA2 v1.6.0 using a 99% percent identity parameter to generate the Amplicon Sequence Variants (ASV). Filtering was performed, in which rare ASVs and singletons, less than 5 reads, were discarded, and were not considered in the final analysis. Finally, the taxonomy was assigned via the UNITE database, which uses the hybrid SINTAX/UTAX algorithm [13]; the sequences were aligned and the taxonomy was assigned to the corresponding ASVs of the 16S and ITS.

### 2.4. Biodiversity Indexes and Statistical Analyses

From the raw data, we obtained 3,352,661 reads ascribable to the ITS and 4,433,595 reads to the 16S. The ASV were further investigated by displaying bar plots of the relative abundance. For each biological component, some biodiversity indices were calculated, as recommended by Morris et al., (2014) [15], using the following R statistical software packages: ‘phyloseq’ [16], ‘microeco’ [17], and ‘vegan’ [18]. The alpha diversity indices used in this work were the Chao1 species richness index and the Shannon (H’) diversity index [19,20]. The distance parameters, seasonality, and biodiversity indices were correlated by means of a Wilcox statistical analysis (*p* < 0.05) in order to assess any significant difference between the three distances (L1, L2, and L3) and the two different seasons (summer and winter). The Bray–Curtis similarity index was used in the PCoA (Principal Coordinates Analysis); the differences between the different functional groups, based on the data obtained in the PCoA, were calculated using the one-way statistical analysis NPMANOVA (*p* value < 0.05).

## 3. Results

### 3.1. Bioinformatic Analysis of Raw Data

Regarding the ITS fungal barcode, the sequencing of 39 samples generated 3,352,661 reads; these were grouped into 1149 total amplicon sequence variants (ASVs). From these, 219 denovo chimeras and singletons were removed for a total of 930 final fungal validated ASVs. For the 16S bacterial barcode, the sequencing of 39 samples generated 4,433,595 reads; these were grouped into 3973 total amplicon sequence variants (ASVs). From these, 791 denovo chimeras and singletons were removed for a total of 3182 final bacterial validated ASVs. Overall, 43% of the dataset was represented by fungi and 57% by bacteria.

### 3.2. Taxonomy Structure and Composition

**Figure 2:**
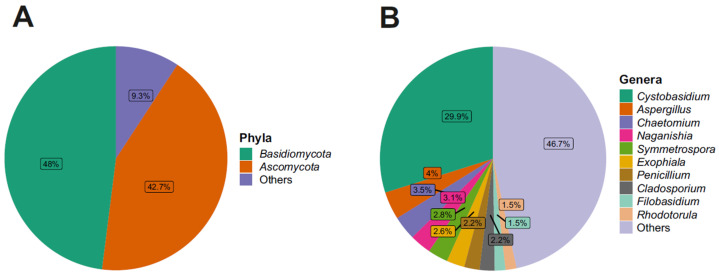
(**A**) Relative abundance of the 2 most abundant phyla. (**B**) Relative abundance of the 10 most abundant genera.

Looking at the taxonomic phylum level, the relative abundance, regarding the fungal component, revealed that two phyla were particularly abundant: *Basidiomycota* (49%) and *Ascomycota* (42%) (Figure 2).

Analyzing the most abundant fungal classes, the *Cystobasidiomycetes* class was found to be most abundant in all three distances from the Concordia Base and in both seasons, noting an abundance of 28% in L1, 44% in L2, and 26% in L3 (Figure S1); furthermore, it can be seen that this fungal class was more abundant in the winter season, where it was present with an abundance of 41%, compared to 23% in the summer season (Figure S2). The next most abundant classes were *Dothideomycetes* and *Eurotiomycetes*, with an abundance of 14% and 10%, respectively, belonging to the phylum *Ascomycota*. In a lower percentage than those listed above, the classes *Tremellomycetes* (8%), *Sordariomycetes* (7%), *Microbotryomycetes* (3%), *Saccharomycetes*, *Leotiomycetes* and *Agaricomycetes* (2%), and *Malasseziomycetes* and *Lecanoromycetes* (1%) were present.

Regarding the fungal orders, *Cystobasidiales*, class *Cystobasidiomycetes* (phylum *Basidiomycota*), was predominant with an abundance value of 30%. The order *Cystobasidiales*, confirming what was observed for its respective class, was the predominant fungal order in all three different distances from the Concordia Base with an abundance of 27% in L1, 38% in L2, and 25% in L3 (Figure S3); as for the two seasons, it was most present in the winter season, where an abundance of 41% can be noted compared to 17% in the summer season (Figure S4). The next most abundant fungal orders were *Pleosporales*, *Eurotiales* with an abundance of 9% and 6%, respectively, belonging to the phylum *Ascomycota*, and the order *Phylobasidiales* with an abundance of 6% belonging to the class *Tremellomycetes* of the *Basidiomycota*. A lower percentage of the orders *Capnodiales* and *Chaetothyriales* (4%), *Sordariales* (3%), *Sporidiobolales*, *Saccharomycetales*, *Hypocreales*, *Tremellales*, and *Helotiales* (2%), and *Malasseziales*, *Glomerellales*, *Erysiphales,* and *Lecanorales* (1%) were found.

**Figure 3:**
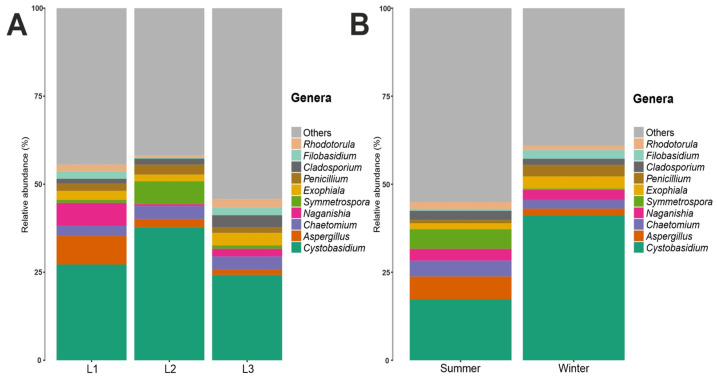
(**A**) Fungal genera relative abundance composition along the three distances (L1, L2, L3). (**B**) Fungal genera relative abundance composition between two seasons.

Regarding the fungal families, *Cystobasidiaceae* (class *Cystobasidiomycetes*, phylum *Basidiomycota*) was the most abundant with an abundance value of 30%; *Cystobasidiaceae*, moreover, was predominant at all three distances and in both seasons, noting an abundance of 27% in L1, 38% in L2, and 25% in L3 (Figure S5). In terms of seasonality, it was more present in the winter season with an abundance of 41% in contrast to the summer season, where it was present with an abundance of 17% (Figure S6). This was followed by fungal families with decreasing abundance values: for example, *Aspergillaceae* and *Filobasidiaceae* (6%), *Didymellaceae* (5%), and *Herpotrichiellaceae*, *Pleosporaceae*, *Chaetomiaceae*, and *Symmetrosporaceae* (3%). Looking to the fungal genera present in the communities analyzed, there was a high degree of heterogeneity, with 38% belonging to the fungal genera found having an abundance of less than 1%, while the genus *Cystobasidium* (family *Cystobasidiaceae*, order *Cystobasidiales*, class *Cystobasidiomycetes*, phylum *Basidiomycota*) proved to be, by far, the most present, with an abundance value of 30%, and was the main representative of the phylum *Basidiomycota* in this study. *Aspergillus* (4%), *Chaetomium* (3.5%), *Naganishia* (3.1%), *Symmetrospora* (2.8%), *Exophiala* (2.6%), *Penicillium* (2.2%), *Cladosporium* (2.2%), *Filobasidium* (1.5%), and *Rhodotorula* (1.5%) (Figure 2) were present in lower percentages. Looking at Figure 3, which shows the 10 most abundant fungal genera between the three distances and the two seasons, it can be seen that *Cystobasidium* was the predominant genus both in the three different distances from the Concordia Base and in both seasons, with statistically significative greater abundance in the winter (41%) than in the summer season (17%).

**Figure 4:**
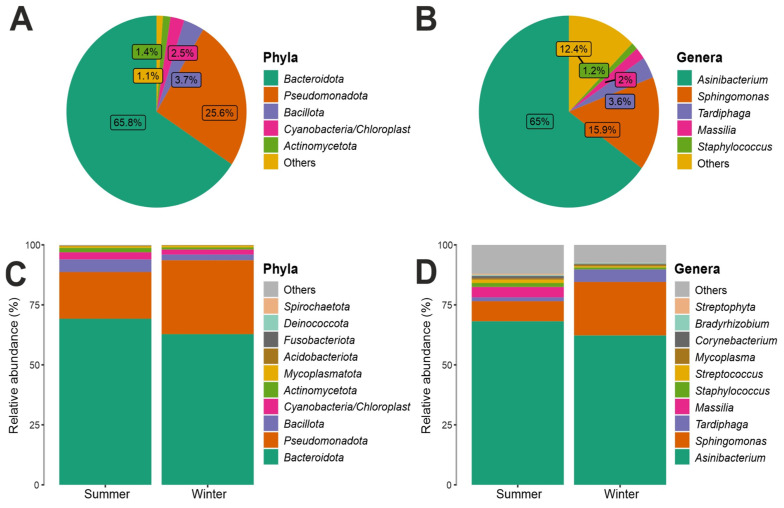
(**A**) Bacterial phyla relative abundance composition. (**B**) Bacterial genera relative abundance. (**C**) Comparison of the topmost abundant phyla between two seasons. (**D**) Comparison of the topmost abundant genera between two seasons.

By examining the 16S rRNA dataset, we observed that the two most abundant results in all ice samples were *Bacteroidota* (68%) and *Pseudomonadota* (26%). *Bacillota* (4%) and *Actinobacteriota* (1%) were found in a lower percentage (Figure 4). At the taxonomic class level, considering the total number of reads obtained in the entire dataset. The class *Sphingobacteriia*, phylum *Bacteroidota*, was predominant with an abundance of 67%, followed by *Alphaproteobacteria* (22%), phylum Proteobacteria. As also shown in Figure S2, which shows the 10 most abundant bacterial classes, the *Sphingobacteria* class was found most prominently in all three distances from the Concordia Base and in both seasons, with an abundance of 63% in L1, 69% in L2, and 68% in L3 (Figure S7); moreover, this bacterial class was present in the summer season, 71%, compared to 64% in the winter season (Figure S8). Similarly, the class *Alphaproteobacteria* was also present in all three distances and in both seasons (Figures S7 and S8), the class *Sphingobacteriia* was present in the winter season, where it had an abundance of 30%, more than in the summer season where it was present with an abundance of 13% (Figure S8). In lower percentages than those listed above, the classes *Betaproteobacteria* (3%) and Bacilli (2%) were found (Figure S8). Figures S9 and S10 shows the 10 most abundant bacterial orders, including the order *Sphingobacteriales*, which was consistently found at all three distances and in both seasons, with a relative abundance of 63% in L1, 69% in L2, and 68% in L3, respectively; at both seasons, it was present with an abundance of 70% in summer compared to winter (64%). The second most abundant order was *Sphingomonadales* with 16% abundance, class *Alphaproteobacteria*, phylum Proteobacteria. The order *Sphingomonadales* was also present in all three distances and in both seasons, with an abundance of 13% in L1, 17% in L2, and 19% in L3 (Figure S9); in both seasons, it was more present, with an abundance of 23%, in winter compared to 9% in summer (Figure S10). With lower abundance values, the orders *Rhizobiales* (5%), *Burkholderiales* (3%), *Bacillales, Actinomycetales*, *Clostridiales*, *Lactobacillales*, *Mycoplasmatales*, *Pseudomonadales*, and *Enterobacteriales* (1%) were found. There was a relative abundance at family level for the bacterial component, considering the total number of reads obtained in the entire dataset. It was found that the family *Chitinophagaceae*, order *Sphingobacteriales*, class *Sphingobacteriia*, phylum *Bacteroidota*, was predominant with an abundance value of 67%. This bacterial family was present at all three distances from the base, with an abundance of 63% in L1, 69% in L2, and 68% in L3 (Figure S11); in terms of seasonality, it was present with an abundance of 70% in the summer season and 64% in the winter season (Figure S12). The next most abundant bacterial family was *Sphingomonadaceae* with an abundance of 16%, belonging to the order *Sphingomonadales*, class *Alphaproteobacteria*, phylum Proteobacteria. The family *Sphingomonadaceae*, similarly to *Chitinophagaceae*, was present at all three distances from Concordia Base and in both seasons, with a higher abundance in the winter season (23%) than in the summer season (9%) (Figures S11 and S12). Figure 4 shows all the genera present in the communities analyzed, it was found that the Genus *Asinibacterium* (family *Chitinophagaceae*, order *Sphingobacteriales*, class *Sphingobacteriia*, phylum *Bacteroidetota*) was predominant with an abundance of 65%, followed by *Sphingomonas* (15.9%), *Tardiphaga* (3.6%), *Massilia* (2%), and *Staphylococcus* (1.2%); furthermore, the boxplots in Figure 4 show the 10 most abundant bacterial genera: a slightly higher abundance was present in summer (68%) than in winter (62%) for *Asinibacterium;* in contrast, *Sphingomonas* was less abundant in summer (9%) than in winter (23%).

### 3.3. Biodiversity Analysis

Analyzing the dataset using the biodiversity indexes, the Chao1 species richness index and the Shannon diversity index (H’) were calculated (Table S2). However, the statistical analysis showed that there were no significant differences either among the three distances or between the seasonalities. Regarding the fungal component, we obtained: L1 (195, 2.15); L2 (175, 1.88); L3 (175, 2.15) for Chao1 and Shannon, respectively, for the distance; summer (184, 2.14) and winter (180, 1.98). Regarding the bacterial component, we obtained: for the distances L1 (555, 2.1); L2 (385, 1.84); L3 (350, 1.74) for Chao1 and Shannon, respectively; for seasonality, we obtained (501, 2.07) for summer and (370, 1.74) for winter for Chao1 and Shannon, respectively.

**Figure 5:**
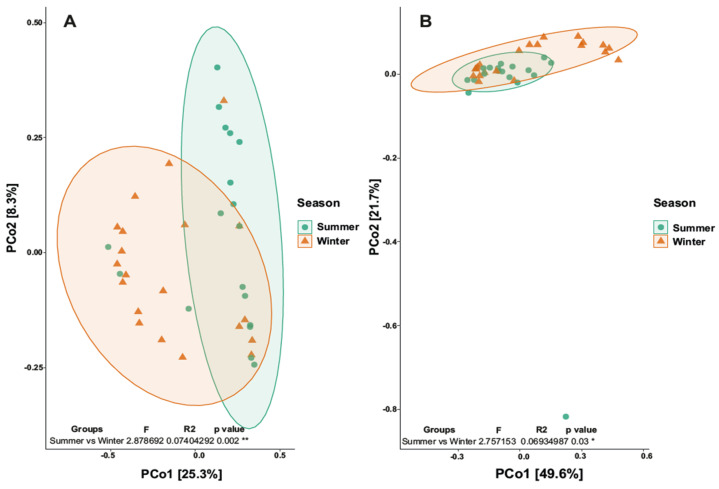
Principal Coordinates Analysis (PCoA). (**A**) Map of the fungal community for the two seasons. (**B**) Map of the bacterial community for the two seasons. * *p* value < 0.05; ** *p* value < 0.01.

Analyzing beta-diversity, the PCoA map relative to the three distances did not reveal separation. The Permanova analysis (L1, L2 and L3) gave a *p*-value of 0.907 (>0.05), revealing no statistical significance. Differently, the PCoA map relative to season revealed a separation between summer and winter (*p* value 0.03; R^2^ 0.07) (Figure 5a). Also, looking at the bacterial component, the PCoA map relative to the three distances did not give separation; in fact, the Permanova analysis gave a *p*-value of 0.89 (>0.05), revealing no statistical significance. On the other hand, the PCoA map revealed a separation between summer and winter (*p* value 0.001; R^2^ 0.074) (Figure 5b).

## 4. Discussion

Glacial habitats pose significant challenges for life, and the Antarctic Polar Plateau’s ice sheet, being the oldest, most isolated, stable, and coldest icy environment on Earth, represents an exceptional case. This study focused on deepening our understanding of the microbial communities, including prokaryotes (using the V4 region of the 16S rRNA gene) and fungi (using the ITS rRNA gene), inhabiting the Antarctic ice shelf of the Polar Plateau. The research was conducted during a 1-year period with monthly sampling, spanning 1 km from the Concordia Research Station. The implementation of the dataset compared to the work of Napoli et al., 2022 [6], was notable, where the 18S rRNA was used to assess the eukaryotic component. From the previous study, it was observed that the eukaryotic component was predominantly composed of fungi. This prompted us to employ the specific ITS rRNA marker for fungi, resulting in the construction of a significantly more comprehensive dataset. Despite the *Ascomycota* being the largest phylum of the fungal kingdom and in contrast with the previous study by Napoli et al., 2022 [6], a greater predominance of the *Basidiomycota* was noted in this study. However, it must be considered that *Basidiomycota* yeasts have a widespread distribution in cold environments, particularly in polar areas [21]. Some works focusing on the study of yeast populations from cold environments have already shown that the presence of basidiomycetoid yeasts, compared to ascomycetoid ones, is prevalent in these environments [22,23]. The *Cystobasidiomycetes* are a heterogeneous group that colonizes a wide range of natural habitats [23,24]. Yeasts belonging to this class have been found in aquatic environments of glacial origin in Patagonia [25], ice sheets in Greenland [26], and also in glaciers in Alpine and Apennine [27]. Members of this class have diverse life strategies, including mycoparasites, endophytes, associated lichens, and fungi adapted to the aquatic environment, in both marine and freshwater. Much of the fungal diversity belonging to this class remains understudied. This prevalence of basidiomycete yeasts could reflect the effect of a more efficient adaptation of this group to the selective pressure typical of glacial ecosystems, such as the formation, in some cases, of a polysaccharidic capsule and a thicker wall than in ascomycete yeasts [28,29]. However, it is reasonable to assume that additional (as yet unknown) adaptation strategies could favor basidiomycete yeasts in extreme ecosystems [23]. Many basidiomycetoid yeasts belonging to the order *Cystobasidiales* have been isolated in Antarctica, but also in other cold ecosystems. As reported by some studies, yeasts belonging to the genus *Cystobasidium* are frequently found in Antarctica and many other cold regions. Antarctic species include *Cystobasidium tubakii* and *Cystobasidium ongulense* isolated from Ongul Island, East Antarctica [30], and the Species *Cystobasidium portillonense* isolated from Antarctic marine sediments and shallow water [31]. In addition, yeasts of the genus *Cystobasidium* have also been isolated from a glacier in the Alps [27]. Other strains have been isolated in Greenland, from a cold lake in Argentina, and from the deep sea [32]. These yeasts of the genus *Cystobasidium* isolated in cold environments all share a psychrophilic or psychrotolerant attitude and are able to adapt to the temperature of the habitat. Among *Ascomycota*, the class *Dothideomycetes* was predominant in our dataset; this is also the largest and most diverse fungal class of the phylum. Members of this class are found on all continents, including Antarctica, and are very important to ecosystem health and the global carbon cycle as saprotrophs and degraders of plant biomass [32]. Many tolerate environmental extremes including heat, cold, solar radiation, and desiccation [33,34]. The order *Pleosporales* was particularly represented in this class; Kirk et al., (2008) [35] reported that most of the species found in snow samples from the Greenland ice sheet belonged to this fungal order.

By examining the 16S rRNA dataset, in fact, the most recurrent phyla were *Pseudomonadota* and *Bacteroidota*, confirming what was previously observed in the few available microbiological studies in the this particular peculiar habitat surrounding the Concordia Base [2,36]. *Bacteroideota* has been also reported as the predominant phylum among Antarctic lake bacteria and on the Tibetan plateau [37,38], while *Pseudomonadota* was also found as the second most abundant prokaryotic representative in the Earth’s major habitat types [39,40,41]. These heterotrophic bacteria have often been isolated, indeed, from a range of other extreme environments, such as deep seas, cold habitats, polar soil, and Antarctic ice cores [36,42]. The ecology of *Asinibacterium* still remains undetermined. So far, taxa belonging to this genus have been isolated from powdered donkey milk [43] and uranium-contaminated subsurface sediments [44], suggesting how this microbe might have physiological and genomic characteristics that allow it to function in the presence of contaminants such as heavy metals. Furthermore, this bacterium has also been isolated from the gut of sea bream [45] and identified in sesame seeds [46]. The second most abundant bacterial Genus was *Sphingomonas*; this result appears to be in agreement with previous studies where this genus was found in snow samples from coastal, central, and interior regions of Antarctica [47]. Previous studies conducted on Antarctic prokaryotes found that *Sphingomonas* was one of the bacterial genera present among the airborne communities on the Antarctic continent [48]. A study conducted on the airborne bacterial population at the Concordia Research Station, indeed, reported *Sphingomonas* as predominant [48]; we argue that the possibility of being aerotransported may explain the presence of this bacterium in the samples we herein analyzed.

We also explored the potential effect of sampling under different environmental conditions (i.e., distance from the Concordia Research Station and seasonality). We found that, using dissimilarity matrices, the seasonality was the main driving factor both for bacteria and fungi (*p* value < 0.05), similarly to what was reported by Napoli et al., 2022 [6], while no significant relationship was observed between the community composition among the three different distances (*p* value > 0.05). Regarding the beta-diversity analysis for the seasonality parameter, the results obtained were, on the other hand, in disagreement with the same similar study [6]. In our study, a significant relationship was observed, showing how the two different seasons influence the composition of the microbial community. The observed separation between summer and winter seasons in microbial composition can be attributed to several factors. Firstly, the seasonal variations in environmental conditions, such as temperature and light availability, can have a significant impact on microbial communities. During the summer, the area was much more impacted by human fluxes with intense logistic and scientific activities related to Concordia Station compared to winter, when the external activities were practically absent in the dark and most cold period. This may have a significant influence on the presence of resident and transient microflora. Furthermore, the atmospheric circulation patterns and air masses during different seasons may contribute to the observed separation [49]. Aerial transport of microorganisms through atmospheric circulation has been recognized as an important source of biological inputs to remote locations, including the Antarctic [1]. Changes in wind patterns and atmospheric conditions between summer and winter can influence the dispersal and deposition of microbial propagules, leading to differences in the composition of microbial communities [49]. It is worth noting that despite the lack of significant separation based on distance from the research station, the presence of microbial communities in the sampled ice/snow suggests the potential for long-range transport of microorganisms in the atmosphere. This highlights the importance of considering atmospheric transport as a mechanism for microbial dispersal in remote environments. Overall, our findings emphasize the significant influence of seasonality on the microbial composition in the Antarctic Polar Plateau. 

Finally, this study may serve as a baseline for future research. Firstly, expanding the sampling efforts beyond the immediate vicinity of the Concordia Base would provide a more comprehensive understanding of fungal and bacterial diversity in the surrounding areas. While logistically challenging, such sampling would help capture the full extent of fungal and bacterial communities in the region. Additionally, investigating the airborne microbial communities in Concordia, including fungal spores, would provide valuable insights into the dispersion and potential sources of these microorganisms. Sampling the air in Concordia would require specialized equipment and careful considerations, but it could shed light on the contribution of airborne microbial transport to the local ecosystem.

## 5. Conclusions

Overall, this study provided the first comprehensive description of bacterial and fungal assemblages of ice/snow samples around the Concordia Base Station in the Antarctic Polar Plateau, contributing also to extending our knowledge of the microbial diversity in cold, icy environments. Further future research in such a pristine environment is worth characterizing, especially with regard to the functionality and potential adaptation of these microorganisms to extreme conditions. This work also highlighted the importance of seasonality in shaping microbial assemblages; we were also able to identify the most sensitive taxa to this factor, which may serve to detect human contamination in this remote environment and give critical clues for future exploration missions.

## Data Availability

Raw sequences are available on the NCBI SRA under the bioproject PRJNA991501.

## Supplementary Materials

The following are available online at https://www.mdpi.com/article/10.3390/biology12091193/s1, Figure S1: Fungal Classes along the distances; Figure S2: Fungal Classes along the season; Figure S3: Fungal Orders along the distances; Figure S4: Fungal Orders along the seasons; Figure S5: Fungal Families along the distances; Figure S6: Fungal Families along the Season; Figure S7: Bacterial Classes along the distances; Figure S8: Bacterial Classes along the seasons; Figure S9: Bacterial Orders along the distances; Figure S10: Bacterial Orders along the seasons; Figure S11: Bacterial Families along the distances; Figure S12: Bacterial Families along the season; Table S1: Samples and Metadata; Table S2: Fungal and Bacterial alpha diversity indexes.
