# Supplementary figures and images for: Seasonality Is the Main Determinant of Microbial Diversity Associated to Snow/Ice around Concordia Station on the Antarctic Polar Plateau
Source: Biology (Basel). 2023 Aug 31;12(9):1193. doi: 10.3390/biology12091193 (PMC10525097; doi:10.3390/biology12091193)

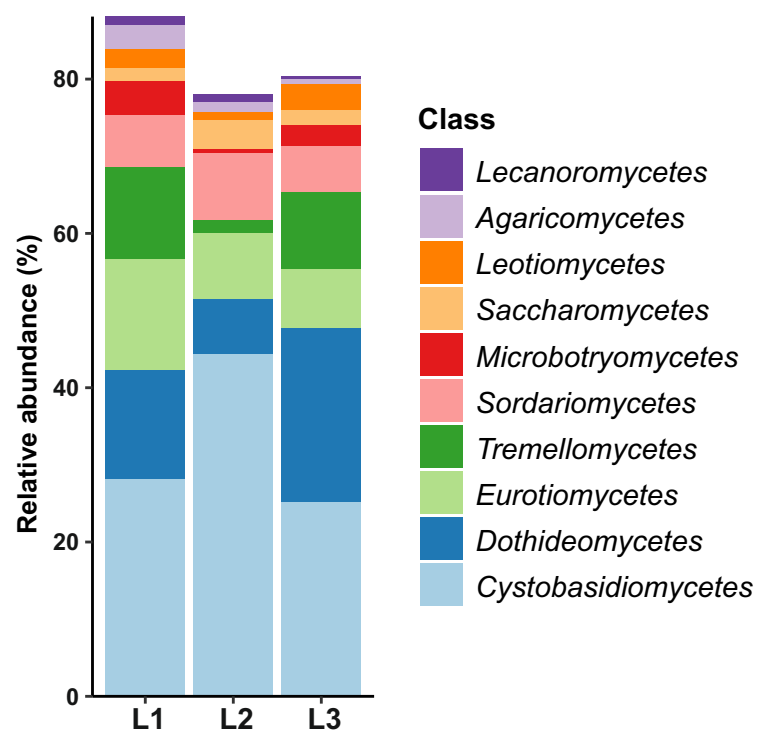

**Figure S1.** Fungal Classes along the distances.

Supplement: Supplementary file 1 [file biology-12-01193-s001.zip › biology-2529015-supplementary/S1.pdf]

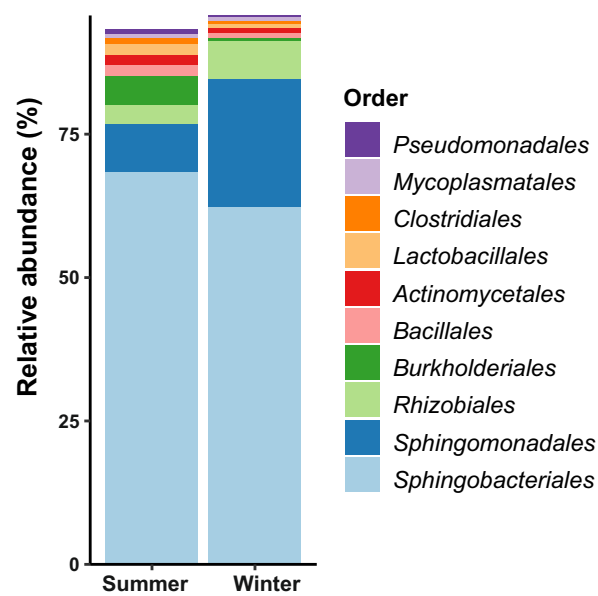

**Figure S10.** Bacterial Orders along the seasons.

Supplement: Supplementary file 1 [file biology-12-01193-s001.zip › biology-2529015-supplementary/S10.pdf]

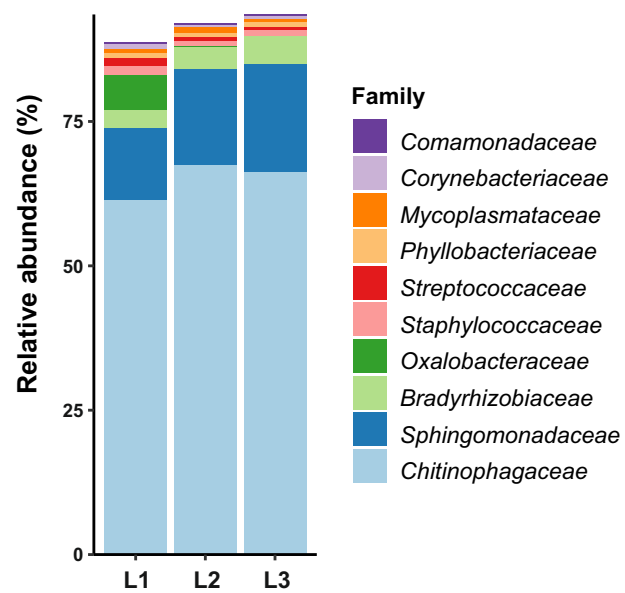

**Figure S11.** Bacterial Families along the distances.

Supplement: Supplementary file 1 [file biology-12-01193-s001.zip › biology-2529015-supplementary/S11.pdf]

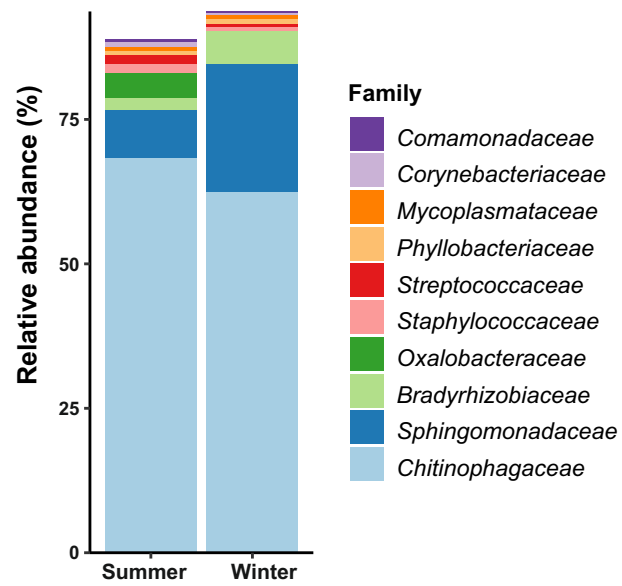

**Figure S12.** Bacterial Families along the seasons.

Supplement: Supplementary file 1 [file biology-12-01193-s001.zip › biology-2529015-supplementary/S12.pdf]

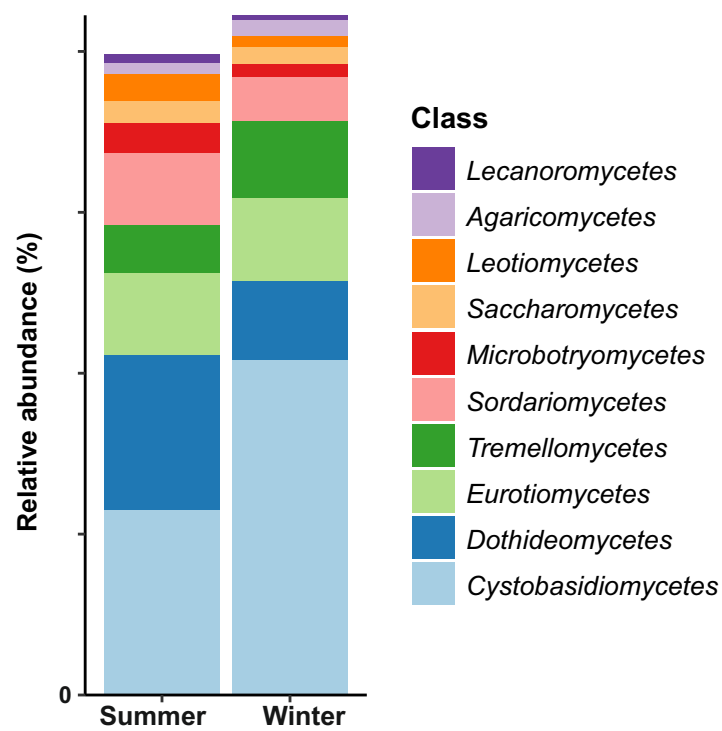

**Figure S2.** Fungal Classes along the seasons.

Supplement: Supplementary file 1 [file biology-12-01193-s001.zip › biology-2529015-supplementary/S2.pdf]

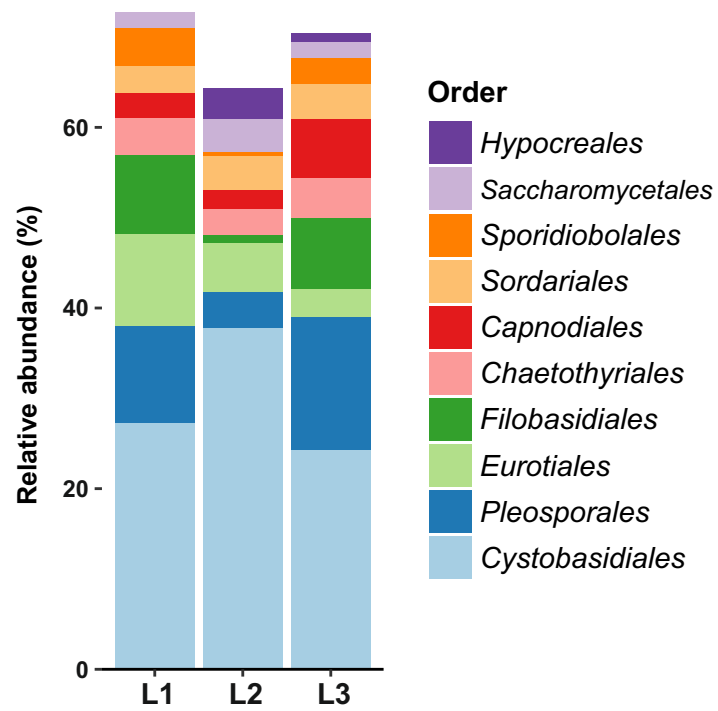

**Figure S3.** Fungal Orders along the distances.

Supplement: Supplementary file 1 [file biology-12-01193-s001.zip › biology-2529015-supplementary/S3.pdf]

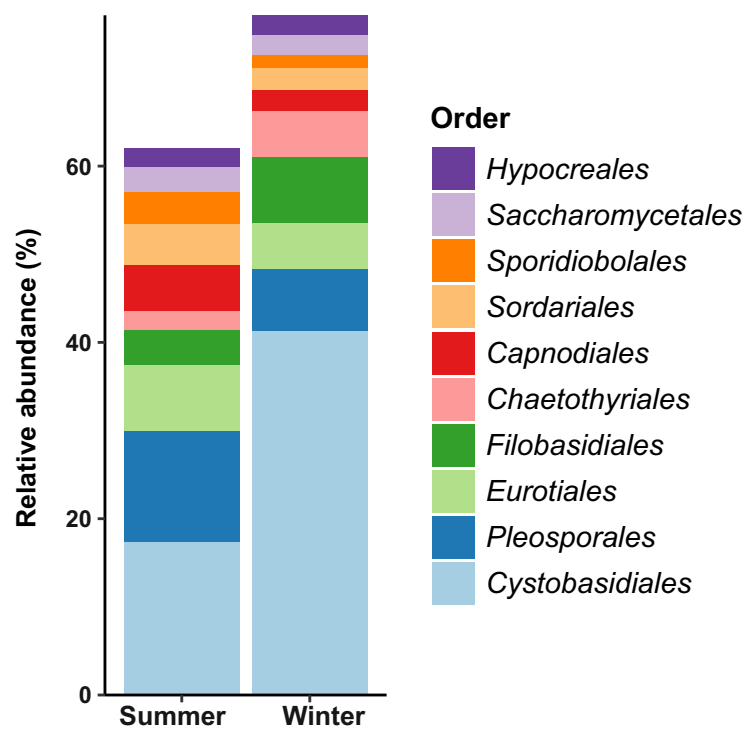

**Figure S4.** Fungal Orders along the seasons.

Supplement: Supplementary file 1 [file biology-12-01193-s001.zip › biology-2529015-supplementary/S4.pdf]

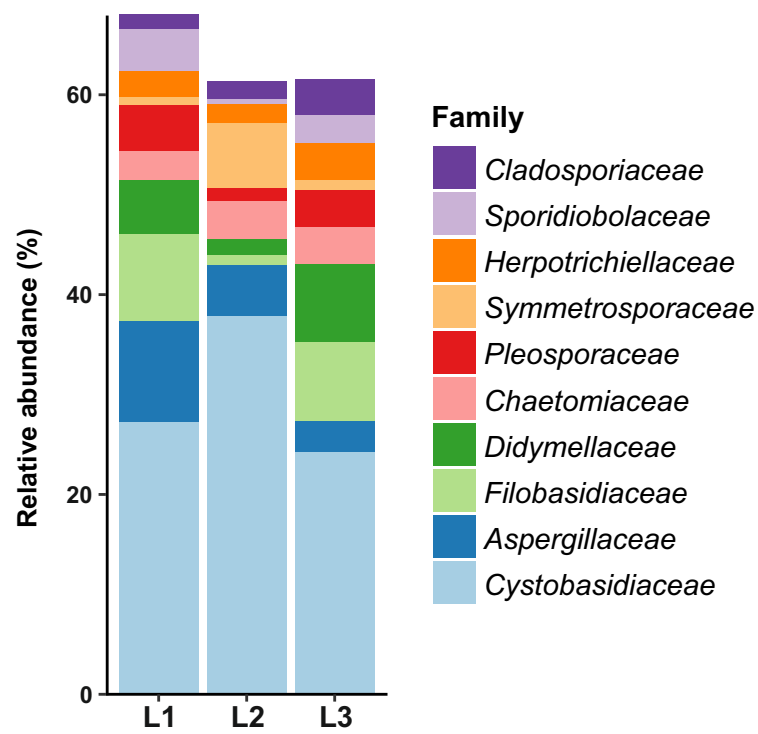

**Figure S5.** Fungal Families along the distances.

Supplement: Supplementary file 1 [file biology-12-01193-s001.zip › biology-2529015-supplementary/S5.pdf]

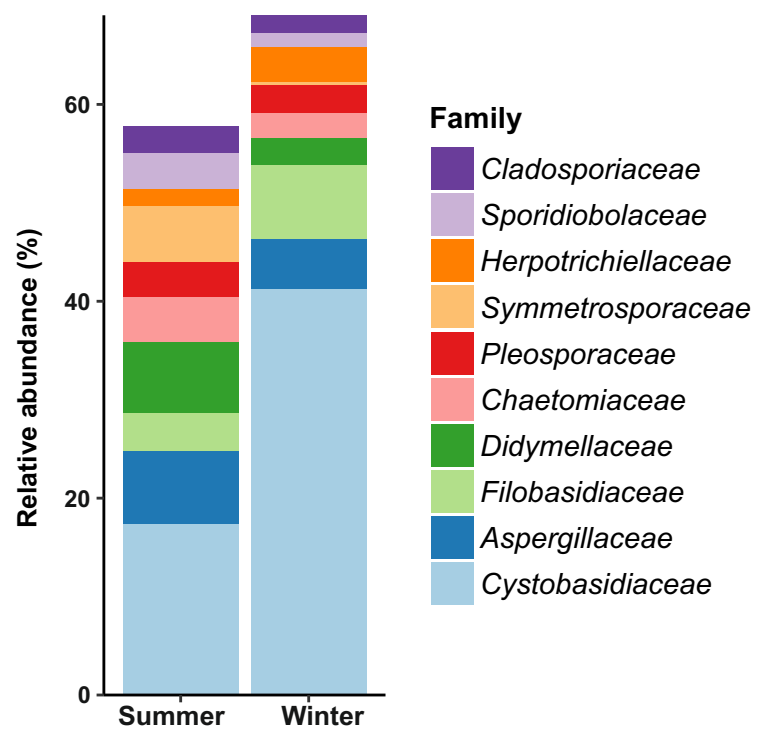

**Figure S6.** Fungal Families along the seasons.

Supplement: Supplementary file 1 [file biology-12-01193-s001.zip › biology-2529015-supplementary/S6.pdf]

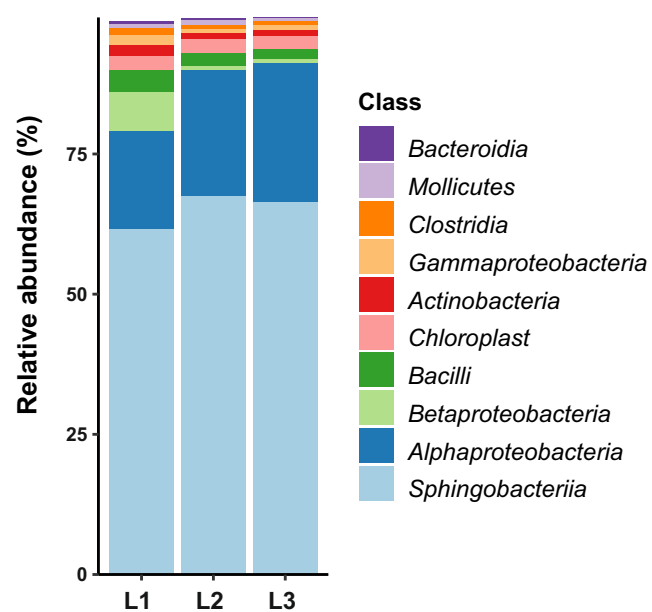

**Figure S7.** Bacterial Classes along the distances.

Supplement: Supplementary file 1 [file biology-12-01193-s001.zip › biology-2529015-supplementary/S7.pdf]

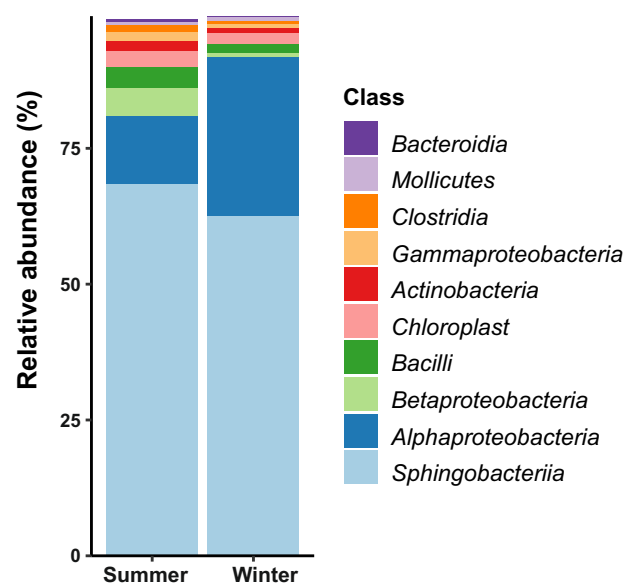

**Figure S8.** Bacterial Classes along the seasons.

Supplement: Supplementary file 1 [file biology-12-01193-s001.zip › biology-2529015-supplementary/S8.pdf]

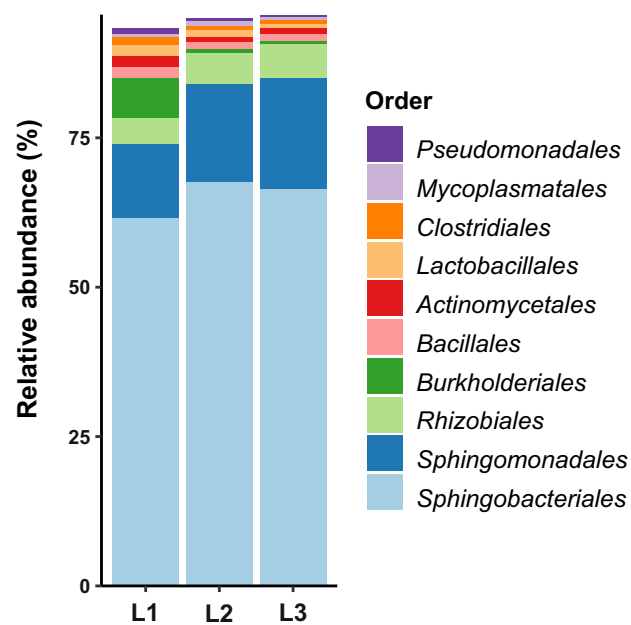

**Figure S9.** Bacterial Orders along the distances.

Supplement: Supplementary file 1 [file biology-12-01193-s001.zip › biology-2529015-supplementary/S9.pdf]
